# An In Vitro Study Measuring the Effects of Circumferential and Near-Circumferential Closed Incisional Negative Pressure Wound Therapy Dressings

**DOI:** 10.7759/cureus.14389

**Published:** 2021-04-09

**Authors:** John P Livingstone, Dylan Singh, Patrick C Murray

**Affiliations:** 1 Orthopedic Surgery, Queen's Medical Center, Honolulu, USA; 2 John A. Burns School of Medicine, University of Hawaii, Honolulu, USA

**Keywords:** ­wound healing, orthopedic surgery, incisional negative pressure wound therapy, sponge, closed incisional negative pressure wound therapy, circumferential, lift off, extremity, perfusion, size

## Abstract

Background

Negative pressure wound therapy (NPWT) and closed incisional negative pressure wound therapy (ciNPWT) have been shown to promote the healing of acute and chronic wounds. Despite the growth in their usage, the mechanism by which they promote healing is not fully understood. Several studies have shown that NPWT results in a combination of microdeformation and macrodeformation, which may promote wound recovery. The macrodeformation forces have raised concerns about circumferential NPWT compressing the extremity and decreasing perfusion distal to the NPWT. The literature on circumferential NPWT is mixed, with some studies showing increased perfusion, while others have shown decreased perfusion. We hypothesized that a near-circumferential ciNPWT dressing applied over intact skin would provide a “lift-off” force rather than a compressive force. We also theorized that as the sponge contracts under negative pressure while in a near-circumferential setting, the dressing will pull on the surrounding skin and tissue, leading to a decrease in the pressure of the extremity. This could potentially translate to improved venous and lymphatic return, increasing perfusion to the tissue beneath the sponge as well as distal to the sponge.

Methods

This study consisted of three separate experiments. The first experiment measured the width and length of a ciNPWT dressing at various negative pressures. The second experiment utilized an in vitro model consisting of an elastic ball and tubing to examine the effects that circumferential and near-circumferential ciNPWT dressings may have on extremity pressure. Varying lengths of ciNPWT dressings were applied to the ball, ranging from 25% circumferential to 100% circumferential. The pressure within the ball was monitored as varying lengths of circumferential dressings were applied at various negative pressures. The third experiment utilized the same model as the second experiment but with a 66% circumferential dressing and various baseline ball pressures to see how extremity pressure may impact the ability of the ciNPWT dressing to alter extremity pressure.

Results

The first experiment demonstrated that a ciNPWT dressing decreased in length and width in a linear fashion as negative pressure was applied. The second experiment revealed that both fully circumferential and near-circumferential dressings resulted in a decrease in the pressure of the elastic ball at lower levels of suction. The greatest decrease in ball pressure was noted with the 66% near-circumferential dressing. With greater suction, however, the pressure within the ball was noted to increase above baseline. The third experiment illustrated that as the baseline pressure of a ball was increased, the dressing had less of an ability to change the pressure of the ball.

Conclusions

These results suggest that near-circumferential and circumferential ciNPWT systems may decrease the pressure of an extremity at certain negative pressures and that compression may be less likely to occur when used on a higher-pressure extremity.

## Introduction

**Figure 1:**
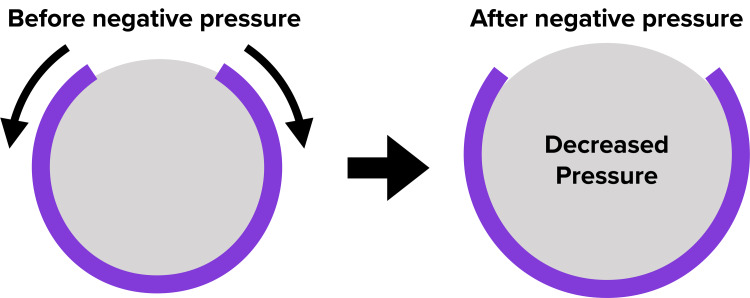
Diagram of the hypothesized "lift-off" force The curved line in purple represents a near-circumferential NPWT sponge, and the gray area represents the extremity that the sponge is applied to. As the sponge contracts under negative pressure, we hypothesized that the sponge would “lift-off” of the extremity, thereby decreasing the pressure of the extremity NPWT: negative pressure wound therapy

A study about the benefits of negative pressure wound therapy (NPWT) was first published in 1997 in a landmark paper by Morykwas et al., in which swine models were found to have a four-fold improvement in perfusion to their wounds with NPWT [1]. This encouraging finding led to several advances in this field. In 2006, closed incisional negative pressure wound therapy (ciNPWT) was introduced, and since its inception, it has continued to evolve with the emergence of newer technology and more indications [2]. Despite their increased usage, the mechanisms behind both negative NPWT and ciNPWT are not fully understood. With all forms of NPWT, it is thought that there is a combination of both microdeformation and macrodeformation at the interface of soft tissue and the NPWT sponge. The microdeformation forces are assumed to increase cell proliferation and angiogenesis through mechanotransduction [3-6]. Several studies have shown increased growth factors such as vascular endothelial growth factor (VEGF) and fibroblast growth factor 2 (FGF2) being associated with NPWT [3,5,7,8]. The macrodeformation forces are thought to be mostly compressive due to the contraction of the sponge under negative pressure [3,6,9,10]. These macrodeformation forces have raised concerns about circumferential NPWT compressing the extremity and decreasing perfusion distal to the NPWT [3]. The verdict on circumferential NPWT in the literature is mixed, with some studies showing increased perfusion, while others have shown decreased perfusion [10-13]. We hypothesized that a near-circumferential ciNPWT dressing, one that does not wrap entirely around an extremity, applied over intact skin would provide a “lift-off” force rather than a compressive force. We also theorized that as the sponge contracts under negative pressure while in a near-circumferential setting, the dressing will pull on the surrounding skin and tissue leading to a decrease in the pressure of the extremity (Figure 1). This could potentially translate to improved venous and lymphatic return, increasing perfusion to the tissue beneath the sponge as well as distal to the sponge. In this study, we utilized an elastic ball as an analog for a human extremity and tested various ciNPWT sponge figurations on the elastic ball.

Our study primarily sought to answer three questions; 1) How do the width and length of the sponge change at various negative pressures? 2) How does a circumferential and near-circumferential sponge affect the pressure of the extremity to which it is applied? 3) How does the baseline pressure of the extremity affect the pressure changes imparted by the near-circumferential sponge?

## Materials and methods

Figure 1 presents a diagram of the hypothesized "lift-off" force.

**Figure 2:**
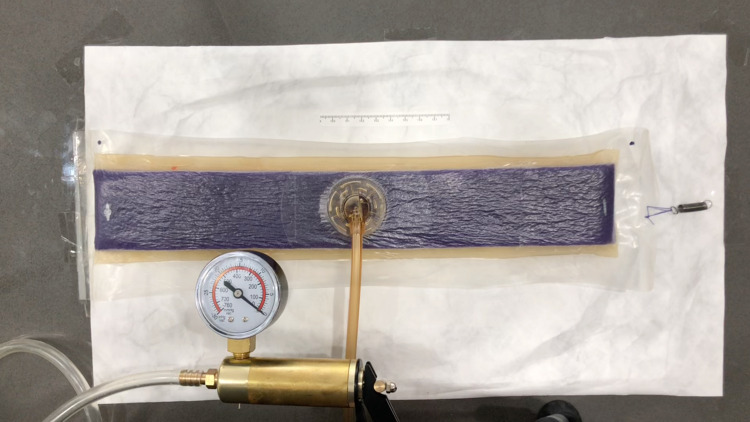
Experiment 1 - measuring the length of the sponge

**Figure 3:**
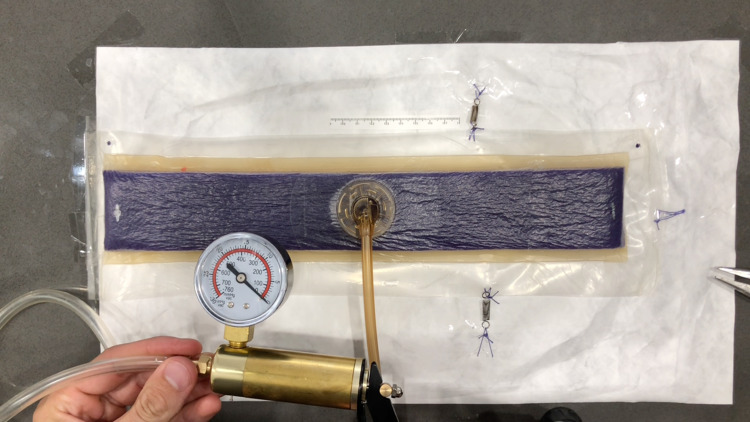
Experiment 1 - measuring the width of the sponge

A PREVENA PLUS^TM ^CUSTOMIZABLE^TM^ dressing by 3M + KCI (3M, Saint Paul, MN) was used for all experiments in this study. The first experiment measured the change in length of the sponge at varying levels of negative pressure. A 35-cm length of the sponge was adhered to the impermeable plastic packaging used for the PREVENA PLUS dressing. The clear adhesive dressings were then applied over the sponge onto the plastic packaging per the manufacturer’s guidelines. In our experience, the NPWT dressing maintains excellent suction without using the additional sealing strips on the short ends of the sponge, and so they were not applied in this study. The plastic was trimmed around the sponge, and the SENSAT.R.A.C.^TM^ Pad (3M, Saint Paul, MN) was applied to the sponge per the manufacturer’s guidelines. The short side of this sponge was secured to the work surface, and the opposite end of the sponge was secured to the work surface with a very sensitive extension spring, which required minimal force to extend (k=50 N/m). This would prevent the wound vac from curling under negative pressure and would allow for a more accurate recording of length (Figure 2). To obtain varying levels of negative pressure exceeding the -125 mmHg capability of the PREVENA PLUS 125 therapy unit, a brake line vacuum pump was used. This vacuum pump was capable of achieving a maximum suction of 760 mmHg. This was secured to the SENSAT.R.A.C. Pad tubing. To measure the changes in length, an iPhone X (Apple Inc., Cupertino, CA) mounted to a tripod was used to record the experiment. The sponge, the brake line vacuum pump negative pressure gauge, and the ruler secured next to the sponge were recorded on video. The still images were then captured from the video at various negative pressure readings ranging from 0 mmHg to -500 mmHg. These images were analyzed using the freely available software, ImageJ. The images were scaled according to the ruler placed next to the sponge and the length of the sponge was measured via ImageJ at various negative pressures. The change in length was then converted to percentage change using Microsoft Excel. In the second part of this first experiment, the same procedure was performed to measure the width of the sponge at various negative pressures. In this part of the experiment, the sponge was secured to the work surface with tape along both of the short sides of the sponge. The long sides of the sponge were secured with sensitive extension springs to prevent the sponge from curling so a more accurate width measurement could be taken (Figure 3).

**Figure 4:**
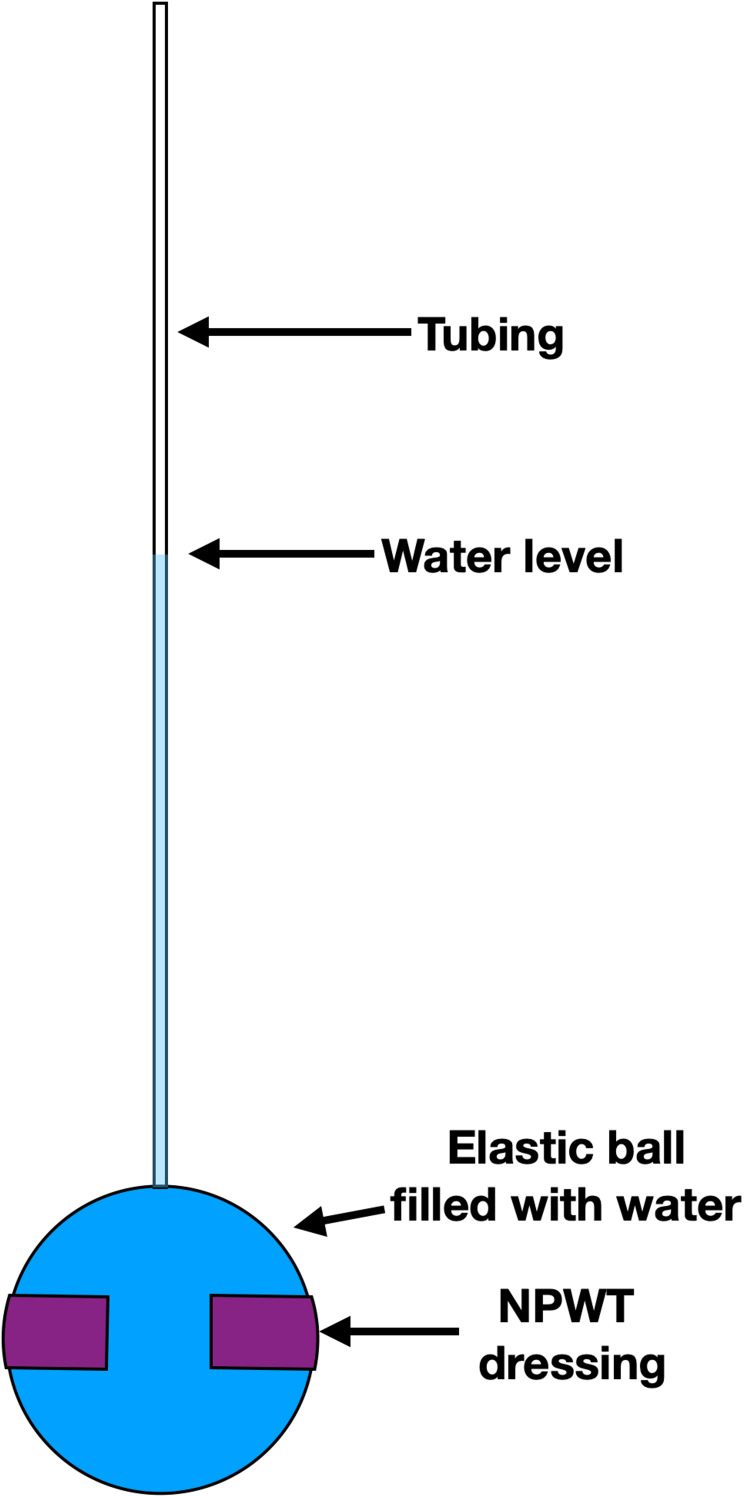
Experiment 2 - the clear vinyl tubing is secured to the ball and filled with water. A tripod is used to record the water level and negative pressure applied to the sponge

**Figure 5:**
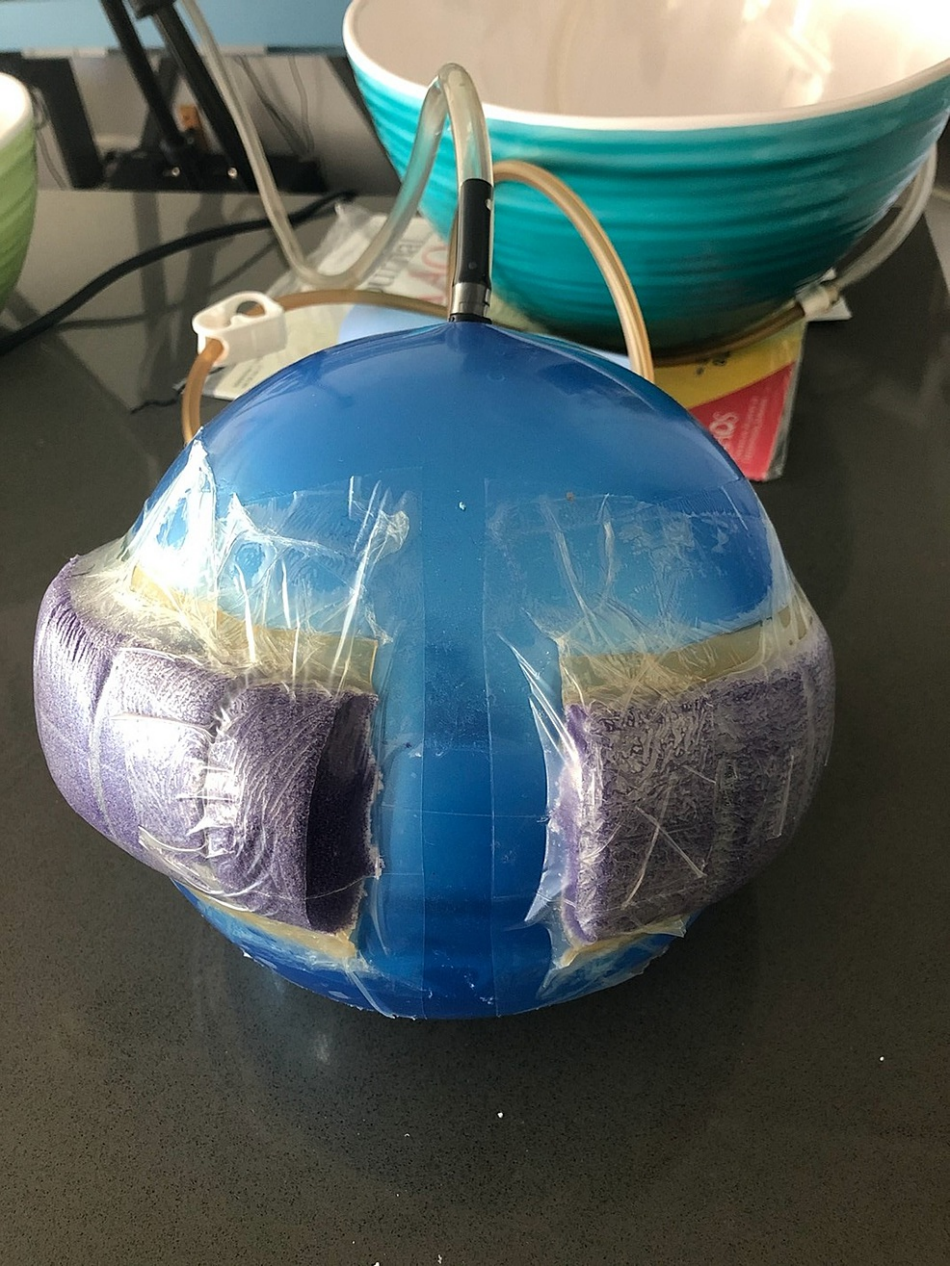
Experiment 2 - 90% circumferential sponge on a ball

The second experiment involved measuring the change in pressure of an elastic ball when a circumferential sponge or different levels of near-circumferential sponges were applied to the ball at various negative pressures. A “bouncy ball” was chosen for these experiments because of its elastic properties and impermeability, which is similar to human skin. A clear vinyl tube was secured into the top of the ball at the inflation port (Figure 4). The clear vinyl tubing was hung above the ball and water was poured through this vinyl tube into the ball until the ball was filled with water. All air bubbles remaining with the ball were removed and the water level could be visualized through the clear vinyl tubing. This water level represented the pressure of the ball in units of cm of water and was noted to be 78 cm for this experiment. The water level could then be monitored at various negative pressures of the sponge. This was accomplished by once again recording a video of the water level within the clear vinyl tubing. The negative pressure gauge of the brake line vacuum pump was included in the frame of the video so it could be analyzed in the same manner as the previous experiments with ImageJ. The scale of these videos was determined with a ruler placed next to the vinyl tubing at the start of the video. The sponge was first placed completely around the ball in a circumferential fashion. This was considered to be 100% circumferential. After the measurements were recorded for this sponge, about 10% of the sponge was cut off and the sponge was resealed with additional adhesive drapes (Figure 5). This was considered to be 90% circumferential. This was repeated for 66%, 50%, and 25% circumferential sponges. Each sponge configuration was tested at various levels of negative pressure ranging from 0 mmHg to -500 mmHg. An interpolation function on Microsoft Excel was used to calculate the estimated negative pressure at which the water level change was 0 mm (intersecting the 0-mm water change along the y axis). This will be referred to as the crossover point since it is at this point where the sponge crosses over from decreasing the pressure of the ball to increasing the pressure of the ball.

The third experiment was performed to study how different baseline extremity pressures affect the changes imparted by the sponge. This experiment was a repeat of the second experiment but with a 66% circumferential sponge and with varying baseline fluid levels as measured through the clear vinyl tubing. A 66% circumferential sponge was used for this experiment after the second experiment showed that a 66% circumferential configuration resulted in the greatest decrease in pressure within the ball. Similar to the second experiment, various levels of negative pressure ranging from 0 mmHg to -500 mmHg were tested. To change the baseline pressure of the ball, additional water was added to the ball until the water level was noted to change significantly and remained stable after several minutes to ensure that all air bubbles had been removed from the ball. The different levels of baseline pressures used were 40 cm, 92 cm, and 128 cm of water. This is equivalent to 29 mmHg, 67 mmHg, and 94 mmHg, respectively. The water levels were recorded and analyzed in the same fashion as in the second experiment.

## Results

**Figure 6:**
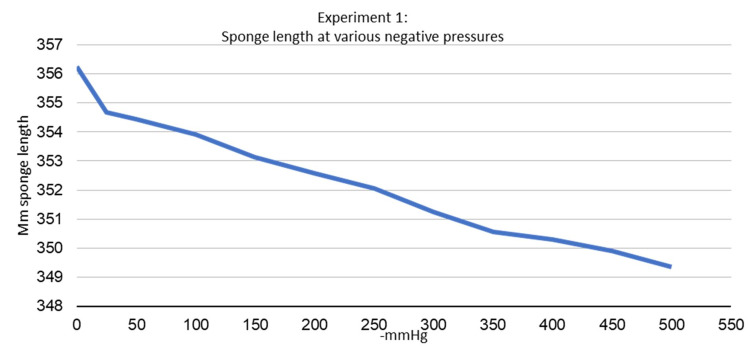
Experiment 1 - sponge length at various negative pressures

**Figure 7:**
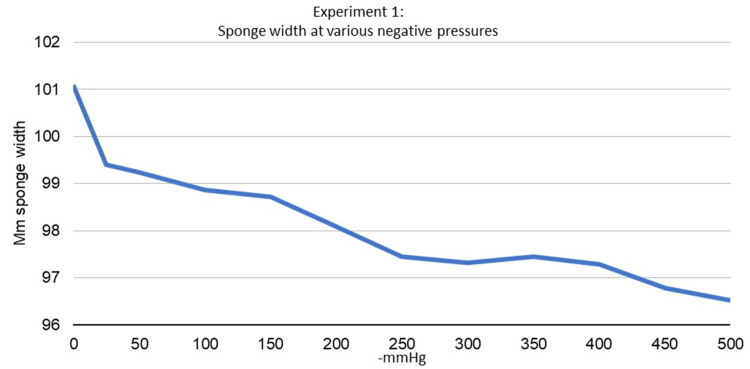
Experiment 1 - sponge width at various negative pressures

The first experiment demonstrated that the sponge decreases in length and width in a nearly linear fashion with negative pressure. As suction increased from 0 mmHg to -500 mmHg, the sponge decreased in both width and length (Figures 6, 7). The maximum decrease in length was noted to be 1.938% at -500 mmHg, and the maximum decrease in width was noted to be 4.515% at -500 mmHg. It was also noted that the sponge edges would curl away from the suction surface under negative pressure in both width and lengthwise directions.

**Figure 8:**
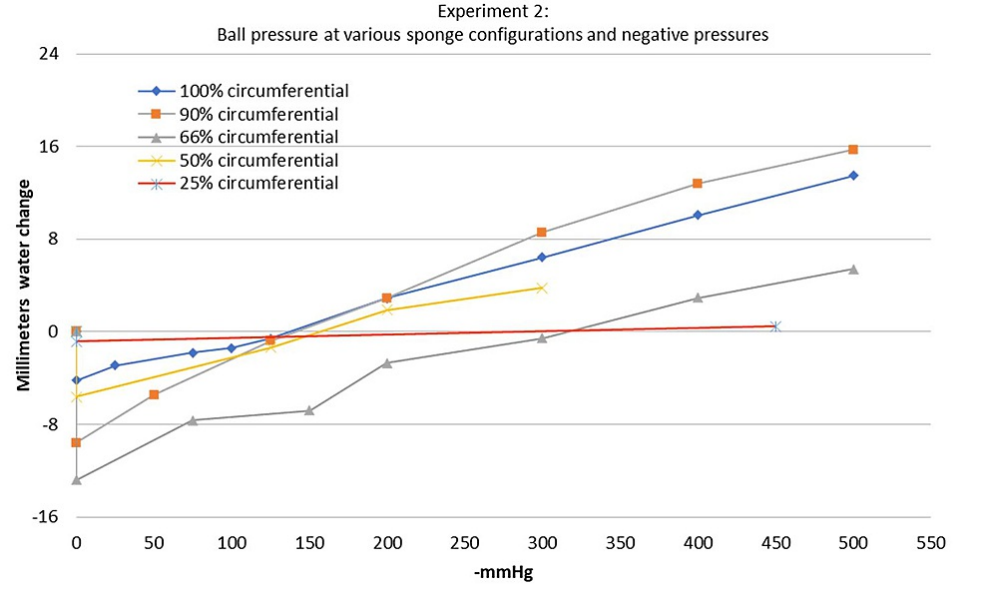
Experiment 2 - ball pressure at various sponge configurations and negative pressures The 0 on the y axis represents the baseline water level before applying negative pressure to the sponge. For this experiment, this baseline water level was 78 cm of water. Note how all of the sponge configurations resulted in an initial decrease in ball pressure and crossed the 0 level on the y axis at varying amounts of negative pressure. The intersection of this line at the 0 on the y axis represents the crossover point where the sponge crosses over from decreasing pressure to increasing pressure in the ball

The second experiment demonstrated that the pressure of the ball first decreased (lower water level) and then increased (higher water level) as greater suction was applied. This was true among all sponge configurations tested from 25-100% circumferential (Figure 8). The water level of the ball was noted to decrease the most before the sponge had completely compressed due to the negative pressure. At this time point, the gauge on the brake line vacuum pump showed 0 mmHg. Although there was a small amount of negative pressure within the system, the vacuum pump gauge was not sensitive enough to read the negative pressure at this time point. The sponge configuration that led to the greatest decrease in ball pressure was the 66% circumferential sponge. The 25% circumferential sponge configuration had almost no effect on the water level with less than 1 mm of water level change at most. Surprisingly, the 100% circumferential sponge configuration still led to an initial decrease in ball pressure and did not begin to compress the ball until the negative pressure reached -137 mmHg. The crossover point when the sponge crossed over from decreasing the pressure of the ball to increasing the pressure of the ball varied with the amount of circumferential sponge being used. This can be visualized by looking at the intersection of the charted line with the 0 mm water change along the y axis (Figure 8). These values are listed in Table 1 as well. The lowest crossover point occurred at -137 mmHg of negative pressure with the 100% circumferential sponge configuration. If the 25% circumferential sponge configuration was excluded since it had nearly no effect on the water level, the highest crossover point occurred at 315 mmHg of suction with the 66% circumferential sponge configuration.

**Table 1:** Experiment 2 - variables The crossover point is defined as the negative pressure at which the sponge crossed over from decreasing the pressure of the ball to increasing the pressure of the ball

Circumferential %	Crossover point (-mmHg)	The greatest decrease in pressure (mm water change)	The greatest increase in pressure (mm water change)
100	137.6433486	-4.204	13.493
90	141.2019491	-9.578	15.704
66	315.2897303	-12.798	5.415
50	156.2557498	-5.616	3.804
25	288.5757807	-0.842	0.471

**Figure 9:**
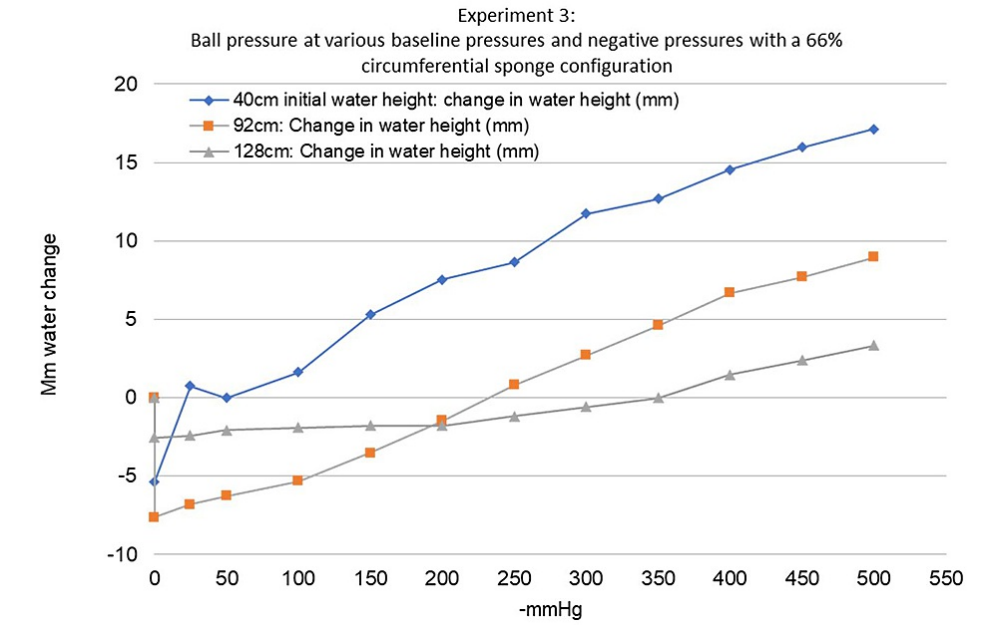
Experiment 3 - ball pressure at various baseline pressures and negative pressures with a 66% circumferential sponge configuration Note that as the baseline pressure increases, the crossover point increases, and the slope of the line decreases

The third experiment demonstrated that changing the baseline pressure of the ball affected the pressure changes imparted on the ball by the sponge (Figure 9). Increasing the baseline pressure of the ball increased the crossover point. Increasing the baseline pressure also decreased the overall ability of the sponge to change the pressure of the ball. This can be visualized by the slope of the lines in Figure 9. The ball with 42 cm of water had a crossover point at only -21.9 mmHg of negative pressure while the ball with 128 cm of water had a crossover point at -350 mmHg of negative pressure. All three baseline pressures tested in this experiment showed a maximum decrease in ball pressure at 0 mmHg while the sponge was still not fully compressed by the negative pressure, similar to the second experiment.

## Discussion

There have been several studies examining the effects of a fully circumferential NPWT system. The first study to examine this was conducted by Kamolz et al. in 2004. This study used a circumferential NPWT system on patients with bilateral partial or full-thickness burns to the hands. The sponge was applied to the more severely burned hand and the other hand received standard dressing changes. Perfusion was recorded daily with angiography, and it was noted that perfusion in the NPWT hand did not decrease like the contralateral hand and was significantly better than the contralateral hand throughout the study [13]. Additional studies on circumferential NPWT systems have been done by Kairinos et al. In their first study, an intracranial pressure monitor was used to measure the pressure changes on a sausage that was sandwiched between two sponges to create a circumferential NPWT system. Negative pressures from -100 mmHg to -500 mmHg were tested on the sausage and the pressure within the sausage was noted to increase as suction increased [14]. This experiment was later repeated on human subjects with hand wounds requiring a circumferential NPWT system. The sponge was applied in a sandwich-like manner around the hand and the intracranial pressure monitor was inserted through the wound bed into the tissue of the hand or finger. Negative pressures from -75 mmHg to -450 mmHg were tested, and similar to the sausage, increased tissue pressures were noted in all subjects at all levels of negative pressure [10]. This experiment was repeated once more, but it utilized radioisotope perfusion imaging to measure the perfusion of healthy hands when placed in a circumferential NPWT system. This study found that perfusion to the hand decreased by an average of 17% in the -125 mmHg group and an average of 40% in the -400 mmHg group [11].

Our study is significantly different from these studies, as we applied the sponge in a strip around the ball, rather than in a sandwich-like manner. Our study was focused on applying the sponge around an extremity, such as the forearm, wrist, lower leg, or ankle, rather than the hand, which is frequently placed between two sponges in a sandwich-like manner. The only study to test a circumferential NPWT dressing on an extremity besides the hand was published in 2020. It measured the SpO_2_ at the fingertip of patients with a fully circumferential NPWT dressing around their upper arm and found no changes in SpO_2_ when negative pressure was applied [12]. Our study also differs from the previously mentioned studies in that we tested the sponge at lower suction levels between 0 mmHg and -75 mmHg. It was during these negative pressures that we observed the greatest decrease in pressure. It is possible that the studies by Kairinos et al. missed these results as they only tested negative pressures between -75 mmHg to -500 mmHg.

This is the first published study to measure the change in width and length of an NPWT sponge at various levels of negative pressure as well as examining its effect when applied in a near-circumferential fashion. Our first experiment demonstrated that the sponge decreased in both width and length as suction increased. The contraction of the sponge in both width and length under negative pressure can lead to the macrodeformation, which is thought to be responsible for some of the positive wound healing associated with NPWT [3,6,9,10]. The sponge length was noted to decrease by a maximum of 1.938% while the sponge width was noted to decrease by a maximum of 4.515%. This finding may be attributed to the customizable nature of the sponge used for this study. The sponge had individual squares of sponge along its length to cut a custom dressing length. There was less contact between the sponge material along the length of the sponge in comparison to the width of the sponge because of these squares. This may decrease the sponge contraction seen along the length of the sponge and account for the difference in the width and length contraction of the sponge in the first experiment.

Our second experiment showed that a “lift-off” force does in fact occur, but only at certain negative pressures. A 66% circumferential sponge provided the greatest decrease in ball pressure as well as the highest crossover point, making it the safest and likely the most effective form of a near-circumferential NPWT system. The default setting for the PREVENA PLUS unit is -125 mmHg, and the crossover point appeared to be at suction levels greater than -125 mmHg for all sponge configurations ranging from 25-100% circumferential. This means that even if a sponge is applied in a 100% circumferential fashion, it may not be compressive at the default -125 mmHg suction.

**Figure 10:**
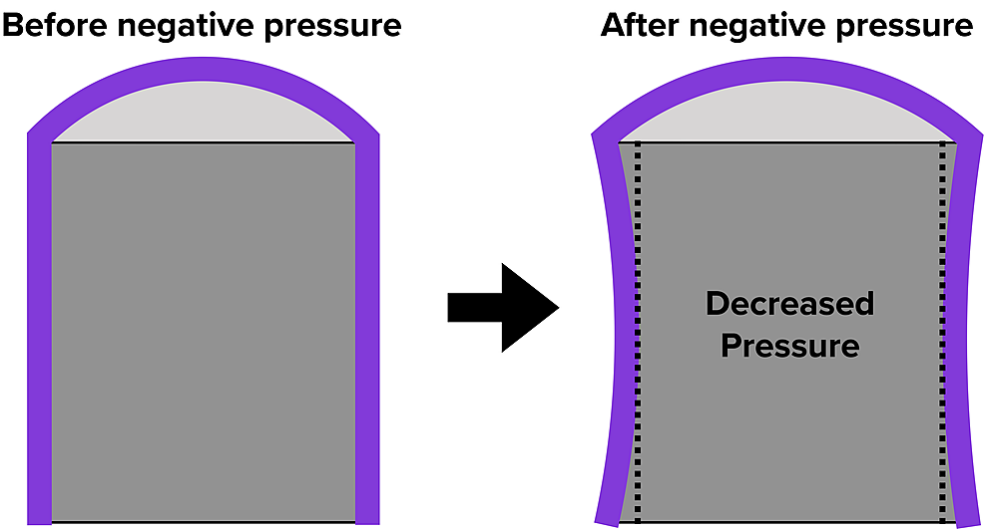
This figure demonstrates the hypothesized “lift-off” force that may occur along the edges of the sponge as the sponge decreases in width when negative pressure is applied to it The gray areas represent the cross-section of an extremity and the purple line represents the sponge. The dotted lines on the right image represent the position of the sponge before negative pressure. Note how as the sponge curls along the edges, the volume of the extremity increases, thereby decreasing the pressure of the extremity

It was a surprising result to see the pressure of the ball decrease in the 100% circumferential sponge configuration. We had hypothesized that the sponge would not be able to “lift” the underlying surface of the ball since it was fully circumferential and that this sponge configuration would lead to compression at all negative pressures. This would also align with previous studies by Kairinos et al. where they saw increased pressures with fully circumferential sponges [10,11]. The initial period of decreased pressure in the ball at lower suctions may have occurred due to two possible reasons. The first reason is that the adhesive drape has some elasticity, which may have allowed the sponge to expand the ball despite the adhesive drape being fully circumferential. The second reason is that there may be some “lift-off” forces along the edges of the sponge. It is important to remember that the sponge decreased in width as well as length in our first experiment. When the sponge decreases in width, it tends to curl around the edges away from the suction surface. This could lift the edges of the ball around the edges of the sponge and contribute to the decreased pressure that was seen at lower suction (Figure 10). This pulling force has also been hypothesized to open up capillary beds and improve perfusion [15]. This phenomenon has been directly visualized via microscopy in animal models as well [16,17]. In light of this, a fully circumferential sponge configuration may increase perfusion and decrease edema, in line with the findings of Kamolz et al. [13].

In the third experiment, the 66% circumferential sponge was tested at various baseline pressures to see how the “lift-off” forces seen in the second experiment would affect a limb with higher pressures such as those seen in a traumatized extremity or compartment syndrome. The 66% circumferential sponge configuration was used since this configuration led to the greatest decrease in ball pressure and had the highest crossover point to prevent compression at most negative pressures. We found that the 66% circumferential sponge had a very low crossover point of 22 mmHg suction at a 40 cm baseline water height and higher crossover points at 232 mmHg and 350 mmHg at 92 cm and 128 cm baseline water heights, respectively. It seems that the higher the pressure the ball has at baseline, the lesser it is affected by the sponge. It also seems that a higher-pressure ball results in a higher crossover point. These higher water levels tested in the third experiment were more consistent with the results from the second experiment although they were not exactly the same. In the second experiment, the crossover point was 315 mmHg at 78 cm of water, meaning that it had a higher crossover point despite having a lower baseline pressure. Although the same make and model “bouncy ball” was used for the third experiment, we were unable to use the exact same ball for the third experiment. This may have led to the differences seen in these two experiments.

To determine which of the baseline water heights tested in the third experiment most closely represent a human extremity, one could compare the pressure of a human extremity with the pressure in the ball. Normal pressures of the lower leg in adults range from 0 to 10 mmHg [18], which is equivalent to 0-13 cm of water. Unfortunately, a pressure of 13 cm of water or less was not achievable in this study since the ball itself was 20 cm in diameter. This means that the lowest possible reading of our in vitro setup was 20 cm of water. The pressures used for this study were closer to the pressures one would expect in compartment syndrome. For a normal diastolic blood pressure of 80 mmHg, a compartment syndrome should be suspected when a compartment pressure is 50 mmHg or greater. This is equivalent to 68 cm of water or greater. Pressures greater than this were tested in the second experiment and in two of the three water levels tested in the third experiment. Even if the pressure of the ball was matched to physiologic extremity pressures, it is not clear if the sponge would have the same effect on the ball as it would have on human tissue. Even if the pressure is matched exactly, the elasticity of the ball will determine the ability of the sponge to change the pressure of the ball. One would predict that a more elastic ball would have a greater ability to expand after the sponge was applied, thereby leading to a greater decrease in pressure. Although the ball used for this experiment was not a perfect extremity analog, the results are clinically relevant since it shows that with a higher baseline pressure, the crossover point occurs with more suction, and the effect of the sponge on the ball pressure is lessened. This means that if there is concern about a near-circumferential sponge compressing an extremity, in theory, the concern should be less for a higher-pressure extremity.

The “lift-off” mechanism described here has not yet been studied in the literature. We hypothesize that this “lift-off” force may increase perfusion beneath the sponge as well as distal to the sponge. By decreasing the internal pressure of the extremity, we hypothesize that venous and lymphatic flow will increase, thereby improving perfusion and reducing edema underneath and distal to the sponge. We theorize that this mechanism can be taken advantage of to improve perfusion and decrease edema in a traumatized extremity, especially in areas where edema is prevalent, such as the lower leg, ankle, and foot. With the PREVENA PLUS CUSTOMIZABLE dressing, there is often leftover sponge and drapes after the incisions are covered with the sponge. These extra supplies could be used to create a near-circumferential sponge proximal to the wound to potentially improve perfusion and decrease edema.

There are several limitations to this study. In the first experiment, the sponge was applied to the plastic packaging from the PREVENA PLUS CUSTOMIZABLE dressing. Since this plastic is not a skin analog, it is not known whether or not the sponge will contract more or less than our experiments when used on human tissue. The extension springs used to keep the sponge flat for width and length recordings may have also led to a decrease in the amount of sponge contraction recorded. Since the elastic balls used for this study could vary significantly from ball to ball based on age, manufacturing tolerances, etc., we chose to only complete the second and third experiments one time each by utilizing the same ball throughout each experiment. The trends seen in these experiments would likely remain the same across different balls, but the changes in ball pressure may differ. The second and third experiments provide insight into the mechanical forces of the sponge on an elastic surface, but the specific changes in the pressure of the ball and crossover points cannot be directly translated into clinical practice. The trends of this study will likely remain true on a human extremity, but we cannot say if the “lift-off” forces will be greater or less than our experimental data when studied on a human extremity. Based on our study, however, a 66% near-circumferential sponge likely has the greatest ability to provide lift-off and has the highest crossover point, making it the safest form of a near-circumferential NPWT system while still providing sufficient “lift-off”.

Future studies should be directed towards replicating these experiments on human or animal extremities. Rather than measuring a water level to determine pressure, continuous compartment pressures could be measured while various sponge configurations and negative pressures are tested. If decreased pressures are seen with near-circumferential sponge configurations in vivo, perfusion and edema could then be measured distal to the sponge. This can be accomplished via angiography [11,13,14,19], thermal imaging [20], and/or volume measurements [21,22].

## Conclusions

This study set out to answer three questions with three different experiments. The first experiment in this study demonstrated that NPWT sponges decrease in both width and length as negative pressure is applied to the sponge. The greater the suction applied to the sponge, the more the sponge decreased in width and length. The second experiment showed that both fully circumferential and near-circumferential NPWT sponges ranging from 25-90% circumferential can decrease the pressure of an elastic ball at lower levels of suction. The greatest decrease in pressure and highest crossover point was seen in the 66% circumferential sponge configuration. The third experiment showed that as the baseline pressure of the ball was increased, the crossover point increased and the sponge had less of an ability to change the pressure of the ball. These three results suggest that near-circumferential NPWT systems may decrease the pressure of an extremity at certain negative pressures and that compression may be less likely to occur when used on a higher-pressure extremity. Since we still do not know the level of negative pressure at which a near-circumferential or circumferential NPWT system may become compressive, we recommend monitoring patients closely after the initial application of this type of dressing.
